# A Practical Model is Equivalent to the BALAD or BALAD-2 Score in Predicting Long-term Survival after Hepatectomy in Chinese Patients with Hepatocellular Carcinoma

**DOI:** 10.7150/jca.51593

**Published:** 2021-01-01

**Authors:** Hua He, Bai Ji, Zhifang Jia, Yangyu Zhang, Xueying Wang, Xuerong Tao, Yahui Liu, Jing Jiang

**Affiliations:** 1 Division of Clinical Research, the First Hospital of Jilin University, Changchun 130021, Jilin Province, China; 2 Department of Epidemiology and Biostatistics, School of Public Health, Jilin University, Changchun 130021, Jilin Province, China; 3 Department of Hepatobiliary and Pancreatic Surgery, the First Hospital of Jilin University, Changchun 130021, Jilin Province, China; Yahui Liu and Jing Jiang. Both authors make an equal contribution to this article.

**Keywords:** Hepatocellular carcinoma, Hepatectomy, Survival, BALAD, BALAD-2

## Abstract

**Aim:** To evaluate the predictive value of the BALAD and BALAD-2 scores on long-term survival after hepatectomy in Chinese hepatocellular carcinoma (HCC) patients and to attempt to establish a more practical or effective model.

**Methods:** A total of 251 HCC patients underwent hepatectomy were recruited. The BALAD and BALAD-2 scores were calculated with total bilirubin, albumin, alpha-fetoprotein, Lens culinaris agglutinin-reactive fraction of alpha-fetoprotein and des-gamma-carboxyprothrombin. The associations of the two scores and their components with the overall survival were analyzed. Finally, three prediction models were explored and constructed.

**Results:** We observed that HCC patients had 5-year survival rates that worsened with increasement of BALAD and BALAD-2 scores. The BALAD and BALAD-2 scores demonstrated fine value in predicting overall survival with Harrell-C statistics of 0.665 (0.618-0.712) and 0.603 (0.554-0.636). After two variables, largest tumor size and BMI, were included in BALAD [0.720 (0.671-0.769)] or BALAD-2 [0.701 (0.649-0.751)] multivariate models, the Harrell-C statistic increased significantly than BALAD (*P*=0.048) or BALAD-2 (*P*<0.001) alone. Taking into account availability and expense, an equivalent BAA-BS model was established based on total bilirubin, albumin, AFP, BMI and largest tumor size. The Harrell-C statistic of BAA-BS model [0.723(0.674-0.772)] was similar to that of BALAD (*P*=0.820) or BALAD-2 (*P*=0.209) multivariate model. And, the continuous net reclassification index and integrated discriminatory improvement were not statistically different. Finally, a nomogram of the equivalent BAA-BS model was constructed to assist surgeons and patients in predicting 5-year survival rates.

**Conclusion:** Both BALAD and BALAD-2 scores were highly suitable for predicting long-term survival after hepatectomy in Chinese HCC patients. A significant increase in predictive efficacy was observed after the addition of largest tumor size and BMI to BALAD or BALAD-2 score. Even if AFP-L3 and DCP are not detected, an equivalent BAA-BS model also obtained an excellent discriminatory performance.

## Introduction

With approximately 466,100 new cases and 422,100 deaths annually 1, liver cancer now has the second largest cancer DALY (disability-adjusted life years) burden in China 2. According to the global data on liver cancer, more than half of the world's new cases and deaths are in China 3. Hepatocellular carcinoma (HCC) is regarded as the main pathological type of liver cancer, comprising 75%-85% of liver cancer cases 4. Currently, curative therapy modalities for HCC, including local ablation, liver transplantation and hepatectomy, are determined mainly by tumor characteristics and liver function 5. Hepatectomy is routinely performed for early-stage HCC, however, the 5-year overall survival rate is just 50% 6. To improve overall survival, it is important to accurately predict long-term prognosis and subsequently apply effective adjuvant strategies after hepatectomy.

In recent years, some miRNAs and lncRNAs have been identified as independent predictors of survival in HCC patients, and the accuracy of prediction has greatly improved 7-11. Taking clinical accessibility into consideration, however, alpha-fetoprotein (AFP) is the most extensively utilized biomarker for predicting the prognosis of HCC 12-14. Subsequently, the combination of Lens culinaris agglutinin-reactive AFP (AFP-L3) 15, 16 with des-gamma-carboxy prothrombin (DCP) 17-19 in addition to AFP obtained an excellent predictive performance^20^. In addition, the deterioration of liver function represented by total bilirubin and albumin is associated with unfavorable postoperative outcomes 21, 22. The BALAD score (the acronym refers to bilirubin, albumin, AFP-L3, AFP and DCP), a model that incorporates the use of the 5 aforementioned objective biomarkers based on the application of conventional cut-off points, was originally developed as a predictor of the survival for patients with HCC in Japan, which has been validated in the UK and Hong Kong 23, 24. After a reassessment using the Japanese data in a continuous format, the BALAD-2 score also offered clear discrimination and has been externally validated in the UK, Germany, and Hong Kong 25, 26. However, etiologies of HCC in China are obviously dissimilar to those of HCC in Japan and European countries the main regions in which the two models were built and validated. Approximately 75%-80% of HCC cases in China are attributable to persistent hepatitis B virus infection, in contrast with the approximately 70% of HCC cases in Japan and European countries mainly attributed to hepatitis C virus infection 27. Moreover, those studies lack specificity for the hepatectomy population because they targeted the total HCC population. Although 27 and 36 patients underwent hepatectomy from Hong Kong, respectively, were involved in two confirmatory studies of BALAD or BALAD-2 24, 26, there is still not sufficient efficacy to justify the feasibility of the two scores in China. Accordingly, this study furtherly evaluated the predictive value of the BALAD score, BALAD-2 score and their components on long-term survival after hepatectomy in Chinese HCC patients.

Because few laboratories at present can simultaneously perform 3 tumor biomarker assays (AFP, AFP-L3, DCP) in China, the accessibility of the two scores is limited. Furthermore, the detection of 5 biomarkers (total bilirubin, albumin, AFP, AFP-L3 and DCP) is bound to require extra costs, so the cost-effectiveness must be considered. Therefore, a more practical model is needed for Chinese HCC patients after hepatectomy to account for clinical operability.

## Materials and Methods

### Subjects

A total of 277 patients were recruited for the study from March 2009 to May 2018 at the First Hospital of Jilin University. Inclusion criteria were as follows: (1) hospitalized for potential hepatectomy; (2) had not undergone any tumor-related treatment before hepatectomy; (3) voluntarily supplied preoperative blood samples; (4) histologically diagnosed with HCC by pathologists. Among the 277 HCC patients, 26 were excluded for one of the following reasons: (1) distant metastasis; (2) positive surgical margins; (3) received anticoagulants such as warfarin; (4) died of perioperative complications; and (5) lost to follow-up at the first interview. Written informed consent was obtained from each patient, and the study protocol was approved by the Ethics Committee of the First Hospital of Jilin University.

### Data collection

Information on general demographic and clinicopathological variables suspected to be risk factors for survival was collected for each patient. Hepatitis B virus (HBV) infection was defined by HBV sero-markers or a history of antiviral HBV treatment 28. Hepatitis C virus (HCV) infection was confirmed by HCV-Ab positivity or a history of antiviral HCV treatment. The largest tumor size and number of tumors were determined from the most recent imaging report prior to hepatectomy. The Child-Pugh class and BCLC stage calculated at the time closest to hepatectomy in each patient were applied. Cirrhosis, vascular invasion, perineurium invasion and histological tumor differentiation were all evaluated according to postoperative pathology.

### Follow-up

Follow-up examinations were carried out 3 months, 6 months, and 1 year after hepatectomy and every year thereafter by specialized staff until death or the last scheduled follow-up. There were three possible follow-up results, as follows. (1) died, the overall survival time was calculated from the date of hepatectomy to the date of death. (2) alive, the overall survival time was calculated from the date of hepatectomy to the date of the latest follow-up. (3) lost to follow-up, the overall survival time was calculated from the date of hepatectomy to the date of the last successful follow-up.

### Measurement of biomarkers

Blood samples were taken from all subjects in 5 mL pro-coagulation tubes the morning before surgery after an overnight fast (at least 8 hours). Serum was separated and stored at -80°C. The magnetic microparticle chemiluminescence immunoassay method was used to measure the concentrations of AFP, AFP-L3 and DCP by a Hotgen MQ60plus automatic immune analyzer (AFP-L3 percentage assay kit, DCP assay kit, Hotgen, Beijing, China). AFP-L3 was extracted by affinity adsorption centrifugation and expressed as the AFP-L3 percentage (AFP-L3%) of total AFP. The interday variation coefficients of the quality control samples were 3.78% for AFP, 3.15% for AFP-L3% and 2.26% for DCP. Total bilirubin and albumin were tested within 12 hours after receiving the blood samples by a HITACHI 7600-210 automatic analyzer. The lab provided daily quality control charts.

### Calculation of BALAD and BALAD-2 scores

The BALAD and BALAD-2 scores were calculated based on the serum levels of the five biomarkers indicating both tumor progression (AFP, AFP-L3%, and DCP) and liver function (total bilirubin and albumin). The tumor marker cut-offs for elevations in AFP, AFP-L3%, and DCP were 400 ng/mL, 15%, and 100 ng/mL, respectively. Total bilirubin was categorized as < 17.1 μmol/L, 17.1-34.2 μmol/L, or > 34.2 μmol/L and assigned 0, 1, and 2 points, respectively, while albumin was categorized as > 35 g/L, 28-35 g/L, or < 28 g/L and assigned 0, 1, and 2 points, respectively. The bilirubin-albumin score was then categorized based on the sum of the 2 values as 0-1, 2-3, or 4 and scored as 0, 1, and 2, respectively. The BALAD score was calculated by simply summing the number of elevated tumor markers and bilirubin-albumin score. The BALAD-2 function was calculated using the following equation: Linear predictor(xb)=0.02*(AFP-2.57) +0.012*(AFP-L3%-14.19) +0.19*(ln(DCP)-1.93)+0.17*(TBIL(μmol/L)^1/2^)-4.50)-0.09*(ALB(g/L)-35.11). The BALAD-2 score was then categorized based on the above BALAD-2 function as ≤-1.74, -0.91 to >-1.74, 0.24 to >-0.91, or >0.24 and scored as 1, 2, 3, and 4, respectively 23, 25.

### Statistical analysis

Continuous variables following a normal distribution are presented as the mean with standard deviation (SD). Otherwise, they were reported as the median with interquartile range (IQR). Categorical variables are shown as frequencies with percentages. The Kaplan-Meier method was used to calculate survival curves and compared by the Log-rank test. The Cox proportional hazard model was used to calculate hazard ratios (HRs) with their 95% confidence intervals (CIs). A multivariate Cox proportional hazard model was performed and included factors with a *P*-value less than 0.1 in the univariate analysis by the forward LR method. The Harrell-C statistic, net reclassification index (NRI) and integrated discriminatory improvement (IDI) were utilized to evaluate the discriminatory performance of the prediction models. The 'CsChange' and 'PredictABEL' packages of R software were used to compare the Harrell-C statistics of different models and calculate NRI and IDI. A predictive nomogram was constructed, and a calibration plot was used to assess the discrepancy. The time-dependent ROC curve of the nomogram was drawn, and the area under the curve (AUC) was calculated. All analyses were performed using SPSS 25.0, GraphPad PRIM8, or R3.6.1 software. For all tests, a two-tailed *P*<0.05 was considered statistically significant.

## Results

As of March 2020, the median follow-up time was 63.6 months. During the follow-up period, 132 (52.6%) patients died of HCC, and 119 (47.4%) patients were still alive. The 5-year survival rate was 45.2% (95% CI: 38.2%-52.3%). The median survival time was estimated to be 54.8 (95% CI: 46.1-63.5) months.

### Associations of general characteristics with all-cause death

The characteristics and overall survival of the subjects included in our study are shown in Table 1. The majority were male (82.9%), classified as Child-Pugh class A (92.4%), had a solitary tumor (78.9%), and had HBV infections (82.9%). The mean BMI was 22.8, and a high BMI was associated with a reduced risk of death [HR (95% CI): 0.90 (0.85-0.96), *P*=0.002]. The median size of the largest tumor was 4.6 cm, and the risk of postoperative death increased by 14% with each increase of 1 cm in largest tumor size [HR (95% CI): 1.14 (1.09-1.19), *P*<0.001]. Approximately half of the patients had vascular invasion (47.8%), and very few patients had perineural invasion (2.0%). Both vascular invasion and perineural invasion indicated poor overall survival. BCLC stage 0 and A was observed in over 70% of all patients, and a high BCLC stage showed an increased risk of death (Table 1).

### Associations of BALAD, BALAD-2 score and their components with all-cause death

**Figure 1:**
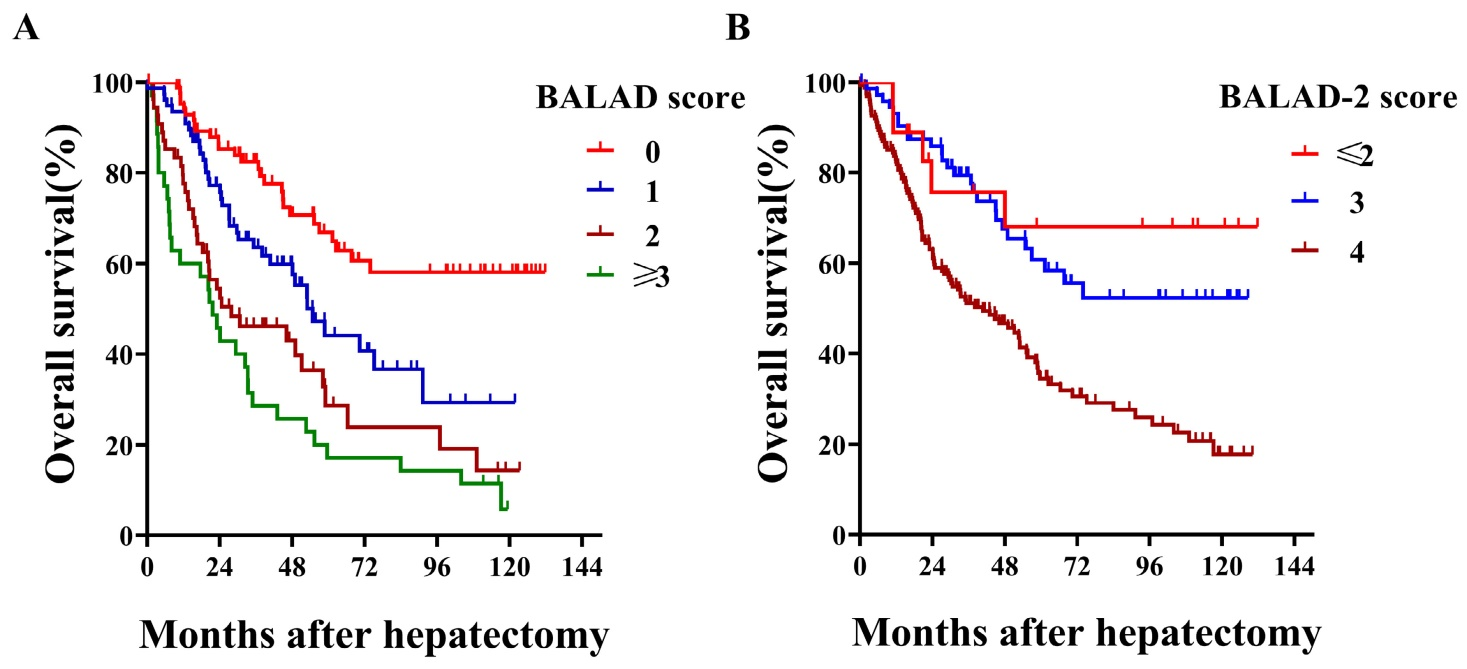
Kaplan-Meier survival curves by BALAD score and BALAD-2 score in all HCC patients

With respect to BALAD score, more than half of the patients were scored as 1 or higher, and no patient was scored as 5 [0 (n=85, 33.9%), 1 (n=77, 30.7%), 2 (n=54, 21.5%), 3 (n=31, 12.3%), and 4 (n=4, 1.6%)]. When comparing different BALAD scores, we observed that HCC patients had 5-year survival rates that worsened with each increase from 0 to ≥3 (66.9%, 44.1%, 28.7% and 17.1%; Log-rank *P*<0.001; Figure 1A). Regarding their predictive value for overall survival, the BALAD score demonstrated a fine Harrell-C statistic with a value of 0.665 (0.618-0.712). Among the different BALAD-2 scores, we found that the 5-year survival rate showed a decreasing trend with each increase from ≤2 to 4 (68.1%, 60.8% and 34.5%; Log-rank *P*<0.001; Figure 1B). Despite the BALAD-2 score being a revision, its Harrell-C statistic was not higher than the BALAD score and was 0.603 (0.554-0.636). In addition, the elevation of each tumor marker (AFP, AFP-L3%, and DCP) and deterioration of liver function (total bilirubin and albumin) significantly indicated poor overall survival (Table 2).

### Multivariate model based on BALAD score or BALAD-2 score

Two multivariate models were built combining the factors of largest tumor size and BMI with BALAD or BALAD-2 score. In this multivariate model, we observed that the risk of postoperative death increased by 48% per each increase of 1 in the BALAD score [HR (95% CI): 1.48 (1.25-1.75), *P*<0.001]. Likewise, the risk of postoperative death rose to 1.65 times per each increase of 1 in the BALAD-2 score [HR (95% CI): 1.65 (1.17-2.34), *P*=0.005]. The Harrell-C statistics of the BALAD and BALAD-2 multivariate models were 0.720 (0.671-0.769) and 0.701 (0.649-0.751), respectively. When comparing the predictive value of different models, a significant increase in Harrell-C statistic was observed after the addition of largest tumor size and BMI to the BALAD (*P*=0.048) or BALAD-2 (*P*<0.001) score, but there was no difference between the BALAD and BALAD-2 multivariate models (*P*=0.244) (Table 3).

### Equivalent BAA-BS model fitted by total bilirubin, albumin, AFP, BMI and largest tumor size

Considering that the Harrell-C statistics of the five biomarkers were approximately 0.6, there was no glaring difference with the Harrell-C statistic of the two scores. We speculated that some of the five biomarkers may have a small contribution, so we tried to combine biomarkers with clinicopathological characteristics to build an alternative model. The Harrell-C statistic of the BAA-BS model [0.723(0.674-0.772)] was similar to that of the BALAD (*P*=0.820) and BALAD-2 (*P*=0.209) multivariate model. The continuous NRI and IDI of the BAA-BS model were not statistically different from those of the BALAD [continuous NRI: 8.92% (-1.08%-13.29%), *P*=0.090; IDI: 0.81% (-1.28%-2.89%), P=0.447] and BALAD-2 [continuous NRI: 12.69% (-1.75%-27.13%), *P*=0.079; IDI: 3.58% (-1.11%-6.04%), *P*=0.103] multivariate models (Table 3).

### Nomogram of the equivalent BAA-BS model

**Figure 2:**
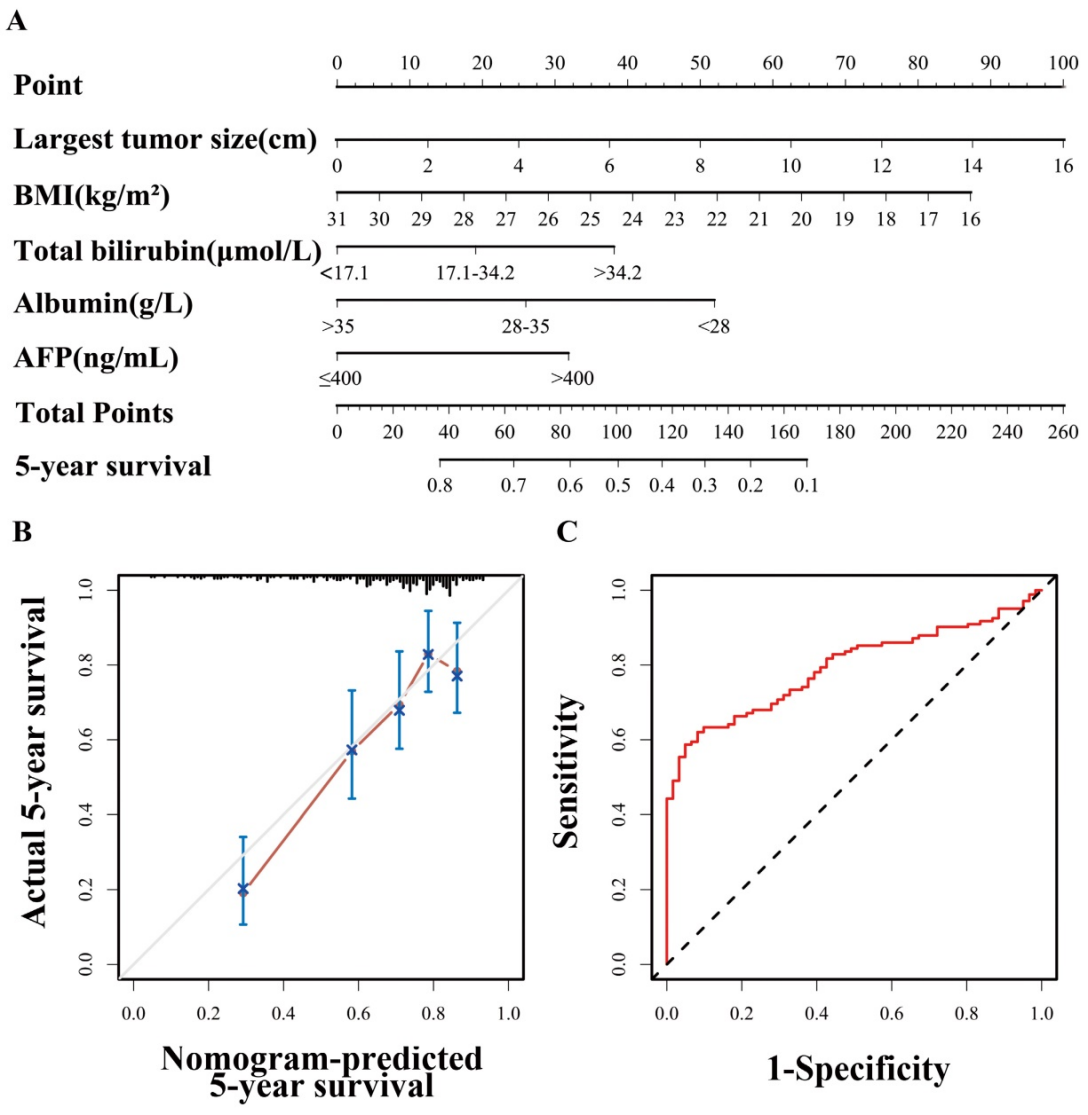
Nomogram of BAA-BS model to predict for 5-year survival probabilities (A). Calibration curve of the nomogram of BAA-BS model between the 5-year nomogram-predicted survival probabilities and actual 5-year survival probabilities (B). Time-dependent ROC curve of the nomogram of BAA-BS model at the 5-year after hepatectomy (C).

A nomogram based on the BAA-BS model is shown in Figure 2A. In the nomogram, each enrolled patient can obtain one individualized score by adding up the points assigned to the five prognostic variables. The projection from the total points (range 0-260) on the scales below predicted the estimated probability of 5-year survival. The calibration plot for 5-year survival probability suggested good consistency between the predicted and observed overall survival probabilities (Figure 2B). Finally, the time-dependent ROC curve suggested that the nomogram possessed good discrimination ability with an AUC of 0.793 (0.727-0.859) (Figure 2C).

## Discussion

Although there have been just a few studies discussing the applicability of the BALAD or BALAD-2 score in the past, there are many differences in nationality, HCC etiology, and treatment methods compared with our HCC population, so a relatively large sample prospective study is urgently needed to illustrate the feasibility and build a sufficient evidence base on the use of the BALAD and BALAD-2 scores in Chinese HCC patients who underwent hepatectomy 29, 30.

This study first focused on Chinese HCC patients after hepatectomy and found that both the BALAD and BALAD-2 scores were highly suitable for predicting long-term survival. This is concordant with a nationwide study in Japan, in which that approximately 75% of the HCC patients had hepatitis C viral infections and the hepatectomy population only accounted for 28.0%, that found that the BALAD score was an effective predictor of overall survival, while approximately 82.9% of the HCC patients had hepatitis B viral infections and all the HCC patients underwent hepatectomy in our study 23. For hepatitis B virus-related HCC patients, a Hong Kong study indicated the versatility of the BALAD score for predicting long-term survival among 27 patients receiving hepatectomy in 198 patients with HCC, in which the advanced HCC accounted for 62.0% 24. On the contrary, our study mainly focused on early HCC in a relatively large sample. Regarding the BALAD-2 score, one Hong Kong cohort externally validated the utility of this score in predicting long-term survival in 36 patients underwent hepatectomy, but it's worth noting that our sample size of hepatectomy population was obviously larger than theirs and our median follow-up time (63.6 months) was also significantly longer than theirs (37 months) 26. With these results in mind, Chinese HCC patients who received hepatectomy with higher preoperative BALAD or BALAD-2 scores should be closely followed up and more comprehensively treated to achieve a prolonged survival period.

With respect to the discriminatory performances of the BALAD and BALAD-2 scores, our study showed that both had a moderate capability to predict all-cause death, but the predictive value of the BALAD-2 score was not superior to that of the BALAD score. This finding is largely consistent with previous research, in HCC patients receiving liver transplantation, except the Harrell-C statistic of the BALAD-2 score differed among studies 31. The dissimilar predictive values of the BALAD-2 score may be attributed to different treatments that the study population received and different detection methods/platforms, which resulted in different fluctuations in the values of the biomarkers. As we know, the BALAD-2 score is calculated with continuous format but still susceptible to fluctuations in the five biomarkers, although a transformation of the variables is performed. These results suggested that the BALAD score could be a more stable predictor of HCC prognosis than the BALAD-2 score across different detection methods or platforms.

Two multivariate models demonstrated excellent discriminatory performances after combining the two easily obtained indicators of largest tumor size and BMI with the BALAD or BALAD-2 score in this study (Table 3 and S1). Tumor size reflects the degree of tumor invasiveness as a part of tumor staging and has been largely adopted in clinical practice to determine patient prognosis and recommend specific treatment for many years 32. In our study, largest tumor size remained an independent risk factor for all-cause death. Regarding BMI, the conclusions of previous studies have been controversial so far. One previous study reported that the 20-year overall survival rate of overweight HCC patients (BMI ≥25.0 kg/m^2^) after hepatic resection was significantly better than that of non-overweight patients 33. Nevertheless, another multicenter study found that underweight (BMI<18.5 kg/m^2^) and overweight (BMI ≥25.0 kg/m^2^) HCC patients appeared to have worse recurrence-free survival and overall survival following liver resection than those who were normal weight 34. Our study showed that the survival rate increased in order from underweight (BMI<18.5 kg/m^2^) to normal weight (18.5 kg/m^2^<BMI<25.0 kg/m^2^) to overweight (BMI ≥25.0 kg/m^2^) patients. After specifically focusing on the normal weight subpopulation, we also observed a decrease in the risk of death per increase of 1 kg/m^2^ in BMI (Table S2). Because nearly 80% of patients were of normal weight in our study, we can only say that HCC patients with higher BMIs in the normal weight range had better long-term survival after hepatectomy than those with lower BMIs. The possible reason for the effect of BMI on overall survival was that patients with a higher BMI in the normal weight range had better a nutritional reserve and metabolic function, which are indispensable for the tumor immune response 35.

In view of the two scores' lack of availability and high cost in China, an equivalent BAA-BS model which is suitable for Chinese HCC patients with hepatectomy was established based on the combination of total bilirubin, albumin, AFP and BMI with largest tumor size. Compared with the previous S-LAD model (Diameter of the largest tumor at time of transplantation, AFP, AFP-L3%, DCP) which was optimized on the basis of BALAD for liver transplantation population 31, liver function indicators were still main predictors in our BAA-BS model, while indicators of liver function were not included in their S-LAD model because of the complete improvement in liver function of HCC patients after liver transplantation. Other than that, in our study, there was a paradox that AFP-L3% and DCP, which had better predictive values than total bilirubin an albumin in the univariate model, were not ultimately entered into the BAA-BS model. After further data analysis, a close correlation between AFP and AFP-L3%, as well as between DCP and largest tumor size, was observed (Spearman's correlation coefficients: 0.701 and 0.753, respectively). As we all know, only one variable among those with strong multicollinearity will be selected for inclusion in the multivariate model, while the other variables will be discarded. Hence, AFP and largest tumor size, replacing AFP-L3% and DCP, were ultimately entered into the BAA-BS model. Regarding the indicators in the model, the largest tumor size is simply and easily obtained, as computed tomography or magnetic resonance imaging must be performed before surgery to determine the tumor site and surgery type. BMI and liver function should also be evaluated as indicators for the patients' ability to tolerate surgery, and AFP is initially detected for the diagnosis of HCC in the Chinese guidelines. In brief, the BAA-BS model is not only accessible but also cost-effective.

Ultimately, we constructed a visual and assessable nomogram on the basis of the equivalent BAA-BS model in which all variables are ordinary indicators and no advanced algorithms are required. Importantly, the discriminatory performance of this nomogram was comparable to that of the two scores' multivariate models, with optimal agreement between the predicted and observed 5-year survival probabilities. Our nomogram offers a good alternative because it does not require detecting AFP-L3 and DCP and can be a powerful assistive tool for surgeons and patients to directly quantify the potential benefit of hepatectomy and indirectly evaluate the risk of all-cause death.

Two major strengths should be mentioned in this study. Our study pays special attention to Chinese HCC patients who underwent hepatectomy, and the extrapolated population for our model is clear and definite. Prognostic nomograms could help both surgeons and patients themselves visually and conveniently calculate and assess the possibilities of survival. However, there are some limitations in our study. All HCC patients included in our study were from only one hospital. In addition, the multivariate model and the equivalent BAA-BS model have not been externally validated. Therefore, more studies with a large sample size are warranted to verify the results.

In conclusion, both the BALAD and BALAD-2 scores were highly suitable for predicting long-term survival after hepatectomy in Chinese HCC patients. A significant increase in predictive efficacy was observed after the addition of largest tumor size and BMI to the BALAD or BALAD-2 score. Even if AFP-L3 and DCP are not detected, an equivalent BAA-BS model including largest tumor size and BMI also obtained an excellent discriminatory performance.

## Supplementary Material

Supplementary tables.

## Figures and Tables

**Table 1:** Associations of general characteristics with all-cause death in all HCC patients

Variable	Classification	Frequency (Percent)	HR (95%CI)	*P* value
Age (years)	<50	80(31.9)	1.00	
	50-59	90(35.9)	0.94(0.61-1.43)	0.760
	≥60	81(32.2)	1.16(0.76-1.78)	0.480
Sex	Male vs Female	208(82.9) vs 43(17.1)	1.23(0.76-2.00)	0.404
BMI (kg/m^2^)	Per increase of 1	22.8(2.6) ^a^	0.90(0.85-0.96)	0.002**
Hepatitis virus infection	Absent	8(3.1)	1.00	
	HBV	208(82.9)	1.14(0.47-2.80)	0.770
	HCV	20(8.0)	0.96(0.33-2.81)	0.939
	HBV+HCV	15(6.0)	0.45(0.13-1.56)	0.207
Cirrhosis	Present vs Absent	158(62.9) vs 93(37.1)	1.13(0.79-1.62)	0.495
Portal hypertension	Present vs Absent	53(21.1) vs 198(78.9)	1.28(0.84-1.96)	0.245
Prothrombin time (s)	>13 vs ≤13	32(12.7) vs 219(87.3)	1.17(0.73-1.89)	0.516
Platelet (10^9^/L)	≥125 vs<125	169(67.3) vs 82(32.7)	1.21(0.84-1.76)	0.314
Child-Pugh class	B vs A	19(7.6) vs 232(92.4)	2.15(1.27-3.62)	0.004**
Largest tumor size(cm)	Per increase of 1	4.6(2.9,7.0)^ b^	1.14(1.09-1.19)	<0.001**
Number of tumors	Multiple vs Solitary	53(21.1) vs 198(78.9)	1.22(0.81-1.82)	0.338
Histological tumor differentiation	Well	26(10.4)	1.00	
	Moderate	135(53.8)	1.26(0.72-2.22)	0.413
	Poor	90(35.9)	1.50(0.84-2.68)	0.170
Vascular invasion	Present vs Absent	120(47.8) vs 131(52.2)	1.83(1.29-2.58)	0.001**
Perineural invasion	Present vs Absent	5(2.0) vs 246(98.0)	2.61(1.06-6.41)	0.037*
BCLC stage	0	15(6.0)	1.00	
	A	171(68.1)	2.93(1.02-9.27)	0.038*
	B	35(13.9)	3.17(1.04-10.70)	0.044*
	C	30(12.0)	6.02(3.02-13.27)	<0.001**

*P < 0.05, **P < 0.01; ^a^ Mean (SD), ^b^ Median (IQR); BMI: body mass index; HBV: hepatitis B virus; HCV: hepatitis C virus; BCLC stage: Barcelona Clinic Liver Cancer stage

**Table 2:** Associations of BALAD, BALAD-2 and their components with all-cause death in all HCC patients

Parameters	Classification	N	5-year survival (%)	Median survival (months)	Log-rank *P* value	HR (95%CI)	Harrell-C statistic (95%CI)
BALAD score	0	85	66.9	NR	<0.001*	1.66(1.42-1.94)	0.665(0.618-0.712)
	1	77	44.1	53.0			
	2	54	28.7	27.9			
	≥3	35	17.1	21.7			
BALAD-2 score	≤2	18	68.1	NR	<0.001*	2.06 (1.47-2.89)	0.603(0.554-0.636)
	3	73	60.8	NR			
	4	160	34.5	40.8			
AFP (ng/mL)	≤400	168	53.9	70.4	<0.001*	2.13(1.51-3.00)	0.588(0.545-0.631)
	>400	83	26.8	29.4			
AFP-L3(%)	≤15	194	50.8	61.3	0.006*	1.67(1.15-2.42)	0.540(0.501-0.579)
	>15	57	25.4	35.2			
DCP (ng/mL)	≤ 100	139	51.8	62.5	<0.001*	1.98(1.40-2.79)	0.605(0.562-0.648)
	> 100	112	35.6	29.7			
Total bilirubin (μmol/L)	< 17.1	133	54.2	73.9	<0.001*	1.67(1.29-2.17)	0.576(0.529-0.623)
	17.1-34.2	99	39.4	45.1			
	> 34.2	19	12.0	24.2			
Albumin (g/L)	>35	211	48.5	58.8	<0.001*	1.84(1.36-2.49)	0.585(0.548-0.622)
	28-35	30	33.1	38.1			
	<28	10	0	10.9			

*P < 0.01; NR: not reach, median survival time could not be estimated as fewer than 50% of patients died; HRs are calculated: per increase of 1 classification

**Table 3:** Multivariate models based on BALAD, BALAD-2 and BAA-BS in all HCC patients

Variable	BALAD multivariate model			BALAD-2 multivariate model			BAA-BS model	
	HR (95%CI)	*P* value		HR (95%CI)	*P* value		HR (95%CI)	*P* value
Largest tumor size (cm) ^a^	1.10(1.04-1.15)	<0.001*		1.12(1.07-1.18)	<0.001*		1.12(1.06-1.17)	<0.001*
BMI (kg/m^2^) ^a^	0.91(0.85-0.96)	0.002*		0.90(0.85-0.96)	0.001*		0.90(0.84-0.96)	0.002*
BALAD score ^b^	1.48(1.25-1.75)	<0.001*						
BALAD-2 score ^c^				1.65(1.17-2.34)	0.005*			
Total bilirubin (μmol/L) ^d^							1.40(1.09-1.81)	0.008*
Albumin (g/L) ^e^							1.59(1.16-2.17)	0.004*
AFP (ng/mL) ^f^							1.76(1.22-2.54)	0.002*
**Harrell-C statistic (95%CI)**	0.720(0.671-0.769)			0.701(0.649-0.751)			0.723(0.674-0.772)	

*P < 0.01; HRs are calculated: [per increase of 1: a; per increase of 1 classification: b(0, 1, 2, ≥3),c (≤2, 3, 4), d(< 17.1, 17.1-34.2, >34.2), e(> 35, 28-35, <28), f(≤400, >400)

## References

[1] Chen W, Zheng R, Baade PD, Zhang S, Zeng H, Bray F (2016). Cancer statistics in China, 2015. CA Cancer J Clin.

[2] Sun D, Cao M, Li H, He S, Chen W (2020). Cancer burden and trends in China: A review and comparison with Japan and South Korea. Chin J Cancer Res.

[3] Bray F, Ferlay J, Soerjomataram I, Siegel RL, Torre LA, Jemal A (2018). Global cancer statistics 2018: GLOBOCAN estimates of incidence and mortality worldwide for 36 cancers in 185 countries. CA Cancer J Clin.

[4] Petrick JL, Florio AA, Znaor A, Ruggieri D, Laversanne M, Alvarez CS (2019). International trends in hepatocellular carcinoma incidence, 1978-2012. Int J Cancer.

[5] Forner A, Reig M, Bruix J (2018). Hepatocellular carcinoma. The Lancet.

[6] Ishizawa T, Hasegawa K, Aoki T, Takahashi M, Inoue Y, Sano K (2008). Neither multiple tumors nor portal hypertension are surgical contraindications for hepatocellular carcinoma. Gastroenterology.

[7] Liu G, Wang H, Fu JD, Liu JY, Yan AG, Guan YY (2017). A five-miRNA expression signature predicts survival in hepatocellular carcinoma. APMIS.

[8] Lin P, Wen DY, Li Q, He Y, Yang H, Chen G (2018). Genome-Wide Analysis of Prognostic lncRNAs, miRNAs, and mRNAs Forming a Competing Endogenous RNA Network in Hepatocellular Carcinoma. Cell Physiol Biochem.

[9] Ma L, Deng C (2019). Identification of a novel four-lncRNA signature as a prognostic indicator in cirrhotic hepatocellular carcinoma. PeerJ.

[10] Yan J, Zhou C, Guo K, Li Q, Wang Z (2019). A novel seven-lncRNA signature for prognosis prediction in hepatocellular carcinoma. J Cell Biochem.

[11] Ding S, Jin Y, Hao Q, Kang Y, Ma R (2020). LncRNA BCYRN1/miR-490-3p/POU3F2, served as a ceRNA network, is connected with worse survival rate of hepatocellular carcinoma patients and promotes tumor cell growth and metastasis. Cancer Cell Int.

[12] Yang SL, Liu LP, Yang S, Liu L, Ren JW, Fang X (2016). Preoperative serum alpha-fetoprotein and prognosis after hepatectomy for hepatocellular carcinoma. Br J Surg.

[13] Shen JY, Li C, Wen TF, Yan LN, Li B, Wang WT (2017). Alpha fetoprotein changes predict hepatocellular carcinoma survival beyond the Milan criteria after hepatectomy. J Surg Res.

[14] Chan MY, She WH, Dai WC, Tsang SHY, Chok KSH, Chan ACY (2019). Prognostic value of preoperative alpha-fetoprotein (AFP) level in patients receiving curative hepatectomy- an analysis of 1,182 patients in Hong Kong. Transl Gastroenterol Hepatol.

[15] Zhang X-F, Yin Z-F, Wang K, Zhang Z-Q, Qian H-H, Shi L-H (2012). Changes of serum alpha-fetoprotein and alpha-fetoprotein-L3 after hepatectomy for hepatocellular carcinoma: prognostic significance. Hepatobiliary & Pancreatic Diseases International.

[16] Saito Y, Shimada M, Utsunomiya T, Morine Y, Imura S, Ikemoto T (2012). Prediction of recurrence of hepatocellular carcinoma after curative hepatectomy using preoperative Lens culinaris agglutinin-reactive fraction of alpha-fetoprotein. Hepatol Res.

[17] Meguro M, Mizuguchi T, Nishidate T, Okita K, Ishii M, Ota S (2015). Prognostic roles of preoperative alpha-fetoprotein and des-gamma-carboxy prothrombin in hepatocellular carcinoma patients. World J Gastroenterol.

[18] Kamiyama T, Orimo T, Wakayama K, Shimada S, Nagatsu A, Yokoo H (2017). Survival outcomes of hepatectomy for stage B Hepatocellular carcinoma in the BCLC classification. World J Surg Oncol.

[19] Tsukamoto M, Nitta H, Imai K, Higashi T, Nakagawa S, Okabe H (2018). Clinical significance of half-lives of tumor markers alpha-fetoprotein and des-gamma-carboxy prothrombin after hepatectomy for hepatocellular carcinoma. Hepatol Res.

[20] Ryu T, Takami Y, Wada Y, Tateishi M, Matsushima H, Mikagi K (2017). Double- and Triple-Positive Tumor Markers Predict Early Recurrence and Poor Survival in Patients with Hepatocellular Carcinoma within the Milan Criteria and Child-Pugh Class A. J Gastrointest Surg.

[21] Johnson PJ, Berhane S, Kagebayashi C, Satomura S, Teng M, Reeves HL (2015). Assessment of liver function in patients with hepatocellular carcinoma: a new evidence-based approach-the ALBI grade. J Clin Oncol.

[22] Ma XL, Zhou JY, Gao XH, Tian L, Wu J, Zhang CY (2016). Application of the albumin-bilirubin grade for predicting prognosis after curative resection of patients with early-stage hepatocellular carcinoma. Clin Chim Acta.

[23] Toyoda H, Kumada T, Osaki Y, Oka H, Urano F, Kudo M (2006). Staging hepatocellular carcinoma by a novel scoring system (BALAD score) based on serum markers. Clinical gastroenterology and hepatology: the official clinical practice journal of the American Gastroenterological Association.

[24] Chan SL, Mo F, Johnson P, Li L, Tang N, Loong H (2015). Applicability of BALAD score in prognostication of hepatitis B-related hepatocellular carcinoma. J Gastroenterol Hepatol.

[25] Fox R, Berhane S, Teng M, Cox T, Tada T, Toyoda H (2014). Biomarker-based prognosis in hepatocellular carcinoma: validation and extension of the BALAD model. Br J Cancer.

[26] Berhane S, Toyoda H, Tada T, Kumada T, Kagebayashi C, Satomura S (2016). Role of the GALAD and BALAD-2 Serologic Models in Diagnosis of Hepatocellular Carcinoma and Prediction of Survival in Patients. Clin Gastroenterol Hepatol.

[27] El-Serag HB (2012). Epidemiology of viral hepatitis and hepatocellular carcinoma. Gastroenterology.

[28] Tian T, Song C, Jiang L, Dai J, Lin Y, Xu X (2020). Hepatitis B virus infection and the risk of cancer among the Chinese population. Int J Cancer.

[29] Johnson PJ (2017). The BALAD-2 and GALAD Biomarker Models for Hepatocellular Carcinoma. Gastroenterology & hepatology.

[30] Roberts LR (2019). Current Status of the GALAD and BALAD Biomarker Models for Hepatocellular Carcinoma. Gastroenterology & hepatology.

[31] Wongjarupong N, Negron-Ocasio GM, Chaiteerakij R, Addissie BD, Mohamed EA, Mara KC (2018). Model combining pre-transplant tumor biomarkers and tumor size shows more utility in predicting hepatocellular carcinoma recurrence and survival than the BALAD models. World journal of gastroenterology.

[32] Cillo U, Vitale A, Grigoletto F, Farinati F, Brolese A, Zanus G (2006). Prospective validation of the Barcelona Clinic Liver Cancer staging system. J Hepatol.

[33] Itoh S, Ikeda Y, Kawanaka H, Okuyama T, Kawasaki K, Eguchi D (2012). The effect of overweight status on the short-term and 20-y outcomes after hepatic resection in patients with hepatocellular carcinoma. The Journal of surgical research.

[34] Yu JJ, Shen F, Chen TH, Liang L, Han J, Xing H (2019). Multicentre study of the prognostic impact of preoperative bodyweight on long-term prognosis of hepatocellular carcinoma. Br J Surg.

[35] Hotamisligil GS (2006). Inflammation and metabolic disorders. Nature.

