# Supplementary material for: A Practical Model is Equivalent to the BALAD or BALAD-2 Score in Predicting Long-term Survival after Hepatectomy in Chinese Patients with Hepatocellular Carcinoma
Source: J Cancer. 2021 Jan 1;12(5):1474–82. doi: 10.7150/jca.51593 (PMC7847645; doi:10.7150/jca.51593)
Supplement: Supplementary file 1 — Supplementary tables. [file jcav12p1474s1.pdf]

Table S1 Multivariate models based on BALAD, BALAD-2 and BAA-BS in hepatitis B virus-related HCC patients (N=208)

Variable	BALAD multivariate model		BALAD-2 multivariate model		BAA-BS model	
	HR (95%CI)	*P* value	HR (95%CI)	*P* value	HR (95%CI)	*P* value
Largest tumor size (cm) [a]	1.09(1.03-1.15)	0.002**	1.12(1.07-1.18)	＜0.001**	1.11(1.05-1.17)	＜0.001**
BMI (kg/m^2) [a]	0.91(0.85-0.97)	0.006**	0.90(0.84-0.97)	0.004**	0.90(0.84-0.97)	0.006**
BALAD score [b]	1.51(1.25-1.82)	＜0.001**				
BALAD-2 score [c]			1.57(1.07-2.31)	0.022*		
Total bilirubin (μmol/L) [d]					1.47(1.10-1.96)	0.009**
Albumin (g/L) [e]					1.79(1.26-2.55)	0.001**
AFP (ng/mL) [f]					1.95(1.33-2.87)	0.001**
**Harrell-C statistic (95%CI)**	0.721(0.668-0.774)		0.693(0.638-0.748)		0.722(0.669-0.775)	

*P < 0.05, **P < 0.01; HRs are calculated: [per increase of 1: a; per increase of 1 classification: b(0, 1, 2, ≥3),c (≤2, 3, 4), d(< 17.1, 17.1–34.2, >34.2), e(> 35, 28-35, <28), f(≤400, > 400)]

Table S2 Multivariate models based on BALAD, BALAD-2 and BAA-BS in normal weight HCC patients ($18.5\ kg/m^2 < BMI < 25.0\ kg/m^2$) (N=195)

Variable	BALAD multivariate model		BALAD-2 multivariate model		BAA-BS model	
	HR (95%CI)	*P* value	HR (95%CI)	*P* value	HR (95%CI)	*P* value
Largest tumor size (cm) [a]	1.12(1.06-1.18)	<0.001**	1.14(1.08-1.20)	<0.001**	1.14(1.08-1.20)	<0.001**
BMI (kg/m^2) [a]	0.88(0.77-0.98)	0.034*	0.86(0.76-0.98)	0.023*	0.85(0.75-0.96)	0.011*
BALAD score [b]	1.40(1.15-1.72)	0.001**				
BALAD-2 score [c]			1.54(1.03-2.31)	0.039**		
Total bilirubin (μmol/L) [d]					1.30(1.03-1.74)	0.035*
Albumin (g/L) [e]					1.68(1.18-2.40)	0.004**
AFP (ng/mL) [f]					1.50(1.09-2.27)	0.024*
**Harrell-C statistic (95%CI)**	0.716 (0.659-0.773)		0.707 (0.650-0.764)		0.718 (0.659-0.776)	

*P < 0.05, **P < 0.01; HRs are calculated: [per increase of 1: [a]; per increase of 1 classification: [b](0, 1, 2, ≥3),[c] (≤2, 3, 4), [d](< 17.1, 17.1–34.2, >34.2), [e](> 35, 28-35, <28), [f](≤400, > 400)]
